# A red-light gate for a cation channel: The conducting state of channelrhodopsin-1 from *Chlamydomonas augustae*

**DOI:** 10.1016/j.bpj.2026.03.019

**Published:** 2026-03-10

**Authors:** Istvan Szundi, Vera Muders, Chie Funatogawa, David S. Kliger, Ramona Schlesinger, Joachim Heberle

**Affiliations:** 1 Department of Chemistry and Biochemistry, University of California, Santa Cruz, Santa Cruz, California; 2 Freie Universität Berlin, Department of Physics, Genetic Biophysics, Berlin, Germany; 3 Freie Universität Berlin, Department of Physics, Experimental Molecular Biophysics, Berlin, Germany

## Abstract

Channelrhodopsins (ChRs) are light-controlled ion channels that have become indispensable tools in the field of optogenetics. Channelrhodopsin-1 from *Chlamydomonas augustae* (*Ca*ChR1) is a phylogenetically early member of this class of proteins with red-shifted absorption and pronounced photocurrent kinetics, but the exact correlation between its photocycle intermediate states and channel conductivity remains to be elucidated. Here, we use time-resolved optical absorption spectroscopy (TROD) in the nanosecond-to-second range for wild-type (WT) *Ca*ChR1 and its E169Q and D299N counterion variants. Singular value decomposition and global exponential fitting revealed kinetic complexity, suggesting parallel photocycle pathways and isospectral intermediates. The spectral deconvolution method resolved five fundamental spectral forms (K, L, M, N, and R) present in the kinetics. Analysis of their temporal evolution, combined with published electrophysiological data, allowed us to identify the conductive state. Contrary to the dominant model that associates conductivity with the deprotonated M state, we show that a late, red-shifted intermediate state, spectrally similar to the K state and called the O state, is the conductive state. The time evolution of this O state parallels that of the channel current in the WT and is consistent with the reduced conduction in the E169Q and D299N variants. Our findings establish a unified mechanism for channel gating in microbial rhodopsins, where a red-shifted intermediate state controls conduction, and provide a new framework for the rational design of optogenetic tools.

## Significance

This study identifies the long-lived, red-shifted O state as the conducting state of the light-gated ion channel *Ca*ChR1, overturning the long-standing assumption that ion flow occurs during the M state. By combining nanosecond-to-second time-resolved spectroscopy with kinetic modeling, the work disentangles previously unresolved intermediate steps and links them directly to functional ion conduction. These findings provide a unified mechanism for channel opening across microbial rhodopsins and establish a clearer foundation for engineering optogenetic tools with improved precision, efficiency, and wavelength tuning—advancing both basic biophysics and applied neuroscience.

## Introduction

Microbial rhodopsins constitute a large and functionally diverse family of light-sensitive membrane proteins. Their common chromophore, retinal, absorbs visible light and undergoes photoisomerization, initiating conformational changes that drive ion transport or signal transduction (1). Among these proteins, channelrhodopsins (ChRs) occupy a special position: as light-gated ion channels, they directly couple photon absorption to transmembrane conductance (1,2). Since their initial discovery (3), the ChR family has expanded through genome mining and functional characterization of new homologs (4). The unique ability of ChRs to convert light into electrical signals has made ChRs valuable tools in optogenetics (5,6), while also providing tractable model systems for exploring the molecular mechanisms of light-driven ion channel gating (7).

Channelrhodopsin-1 from *Chlamydomonas augustae* (*Ca*ChR1) represents an early evolutionary member of the ChR family and exhibits distinct photochemical properties compared with the canonical *Chlamydomonas reinhardtii* homologs (8). While both channelrhodopsins conduct cations, albeit with different specificities, *Ca*ChR1 displays a red-shifted absorption maximum in the visible range (λ_max_ = 520 nm in *Ca*ChR1 (8) vs. λ_max_ = 470 nm in *Cr*ChR2 (9)) and characteristic current kinetics with pronounced adaptation under continuous illumination, which represent improved features for optogenetic applications.

**Figure 1:**
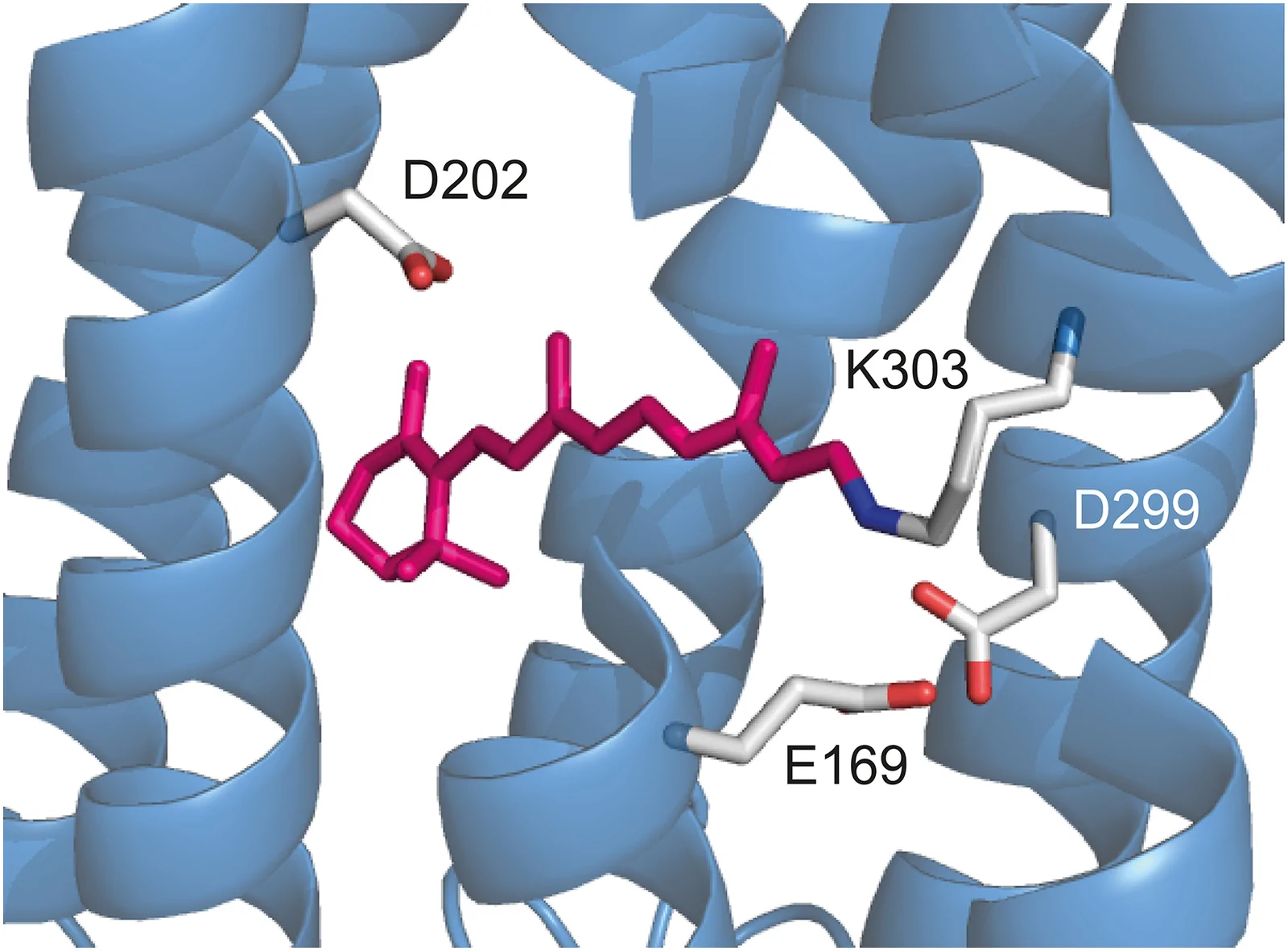
Retinal binding pocket of *Ca*ChR1. All-*trans* retinal (*magenta*) forms a Schiff base linkage with K303 on helix G. Carboxylic residues E169 and D299 are potential counterions to the protonated Schiff base, whereas D202 serves as the internal proton donor. The *Ca*ChR1 model was generated using the SWISS-MODEL server with the C1C2 structure (PDB: 3UG9 (16)) as a template. Figure replotted from (19).

High-resolution structures of a number of ChRs and their engineered variants have been determined by x-ray crystallography (10,11,12,13,14,15,16) or by single-particle cryoelectron microscopy (11,17,18). Although no experimental structure is yet available for *Ca*ChR1, homology modeling suggests that its overall fold resembles that of the C1C2 chimera (chimera construct of helices A–E of *Cr*ChR1 and helices F and G from *Cr*ChR2). Most residues critical for function in *Cr*ChR2 are conserved in *Ca*ChR1, including K303, which forms the Schiff base linkage to the retinal chromophore, the two putative counterions E169 and D299, and the internal proton donor D202 (Fig. 1).

Time-resolved spectroscopic studies of *Ca*ChR1 have characterized its photocycle over timescales ranging from femtoseconds to seconds (20,21,22,23), yielding a simplified picture of the photocycle. Pulsed photoexcitation of the dark state of *Ca*ChR1 induced an all-*trans* to 13-*cis* isomerization, producing the red-shifted K-like photoproduct. This isomerization is the fastest observed for any microbial rhodopsin (22). It has been reported that the K-like intermediate is followed by the blue-shifted M-like intermediate emerging in two phases within the early *μ*s time range. (20) In the wild-type (WT) protein, the M intermediate reaches its maximal population after approximately 4 ms and subsequently decays in two steps on late ms timescales. This M-like intermediate is accompanied by red-shifted intermediates: a K-like at early times and a weakly absorbing O-like at late times. Notably, intermediates analogous to L or N have not been reported. The K-, M-, and O-like intermediates reported correspond to the P_1_, P_2_, and P_4_ intermediates, respectively, as identified in the initial stages of UV-vis spectroscopy of channelrhodopsins (19,23,24,25).

Electrophysiological measurements have linked the formation and decay of the M intermediate to transient photocurrents on the millisecond timescale (20). The intermediate involved in the channel closure has also been investigated by FTIR spectroscopy (26). This state differs from the dark configuration by backbone rearrangements and a 13-*cis*-configured retinal, which reisomerizes to all-*trans* only after the state decays (19).

Channel currents in *Ca*ChR1 are accompanied by large transient currents that overlap in time with the opening of the channel. These transients are linked to proton movements during Schiff base deprotonation. Mutagenesis studies revealed that the two counterions affect these currents differently: E169Q exchange markedly reduces both outward proton transfer and channel current, whereas D299N exerts only a minor effect on proton transfer but strongly reduces channel current (20).

Whereas UV-vis and resonance Raman spectroscopies (19,27) are sensitive markers for conformational changes of the chromophore retinal, IR spectroscopy (19,26,28,29), and photoacoustic spectroscopy (30) resolve structural changes of the surrounding apoprotein and the associated water molecules with high temporal resolution. In *Ca*ChR1 and *Cr*ChR2, the K state is associated with large protein conformational changes as inferred from FTIR experiments (24,29). Photoacoustic experiments confirmed these findings and quantified the volumetric changes (30) that are induced by the transient protonation of E169 and D299.

In this study, we combine time-resolved UV-vis spectroscopy with kinetic modeling to dissect the photocycle, to project it into the evolution of five fundamental spectral states, and to reveal parallel reaction pathways. We identify a late, red-shifted intermediate state—distinct from the M state—as the principal conducting state. The time evolution of this intermediate state matches the channel current kinetics in the WT protein and is consistent with the reduced current levels displayed by the counterion variants E169Q and D299N. These findings challenge the prevailing model of cation channelrhodopsin gating and offer new perspectives for rationally engineering next-generation optogenetic tools.

## Materials and methods

### Sample preparation

*Ca*ChR1 and its variants were prepared as previously described (31). *Ca*ChR1 samples were stored in an aqueous solution of 20 mM HEPES, 100 mM NaCl (pH 7.4), 0.03% DDM (*n*-dodecyl-β-D-maltopyranoside). WT as well as variants E169Q and D299N in this work identify a truncated form of *Ca*ChR1 (1–352 aa) with a 10xHis tag at the C-terminus.

### Time-resolved optical absorption measurements

Time-resolved optical absorption difference spectra (TROD), post- minus preillumination, were recorded as described earlier (32). The TROD spectra were recorded at logarithmically spaced delay times, three per decade. The kinetic analysis was carried out on data collected in 2–3 days with 80 records per day, averaged for each delay time. The experiments were performed at room temperature using a quartz cuvette of 0.4 cm path length filled with a plastic insert that provided a ∼10 *μ*L observation chamber into which 10 *μ*L of fresh sample was pumped before each photoexcitation to prevent secondary photoreactions.

### Data analysis

The TROD spectra were analyzed with programs written in MATLAB (The MathWorks, Natick, MA) as described earlier (32). The temporal (V) and spectral (U) vectors in the experimental data matrix were separated using singular value decomposition (SVD): *ΔA = U* × *S* × *V*^*T*^. Exponential fitting of the significant temporal vectors yielded the apparent rates, and the amplitude or b-spectra were calculated using the significant spectral vectors, the corresponding significance values in *S*, and the results of the V-vector fit. The b-spectra were converted into intermediate spectra, *In(λ,n)*, of the straight sequential scheme in which the intermediates decay with the apparent rates. The spectra of the sequential intermediates were deconvoluted into spectral forms, *In(λ,n) = E(λ,m)* × *Q(m,n)*, where *E(λ,m)* is the matrix of the spectral forms and *Q(m,n)* is the composition matrix showing the contributions by each spectral form, *m*, to the intermediate spectra. The time evolution of the spectral forms, *C(m,t)*, was calculated as the product of the composition matrix and the matrix of time-dependent concentrations, *C*_*in*_*(n,t)*, of the sequential intermediates, *C(m,t) = Q(m,n)* × *C*_*in*_*(n,t)*.

## Results and discussion

Understanding of the molecular mechanism of the red-shifted natural variant *Ca*ChR1 lags far behind *Cr*ChR2, the most frequently used optogenetic tool. Specifically, the temporal and structural coupling of retinal isomerization, proton transfer, and ion conduction is unclear, as is the conductive nature of the intermediate states. Similarly, the quantitative description of the kinetic coupling between photochemistry and the electrophysiological response is lacking. Thus, we applied TROD spectroscopy (32), which provides superior spectral information compared with the traditional single-wavelength approach (20).

High-quality spectral data are difficult to obtain on samples that show significant levels of light scattering, such as membrane suspensions and liposomes. The proteins produced are solubilized in DDM, which is optimal for spectroscopic measurements. Some distortion of the kinetics is possible, as in all rhodopsin kinetic studies; however, these are considered to be within tolerable limits.

**Figure 2:**
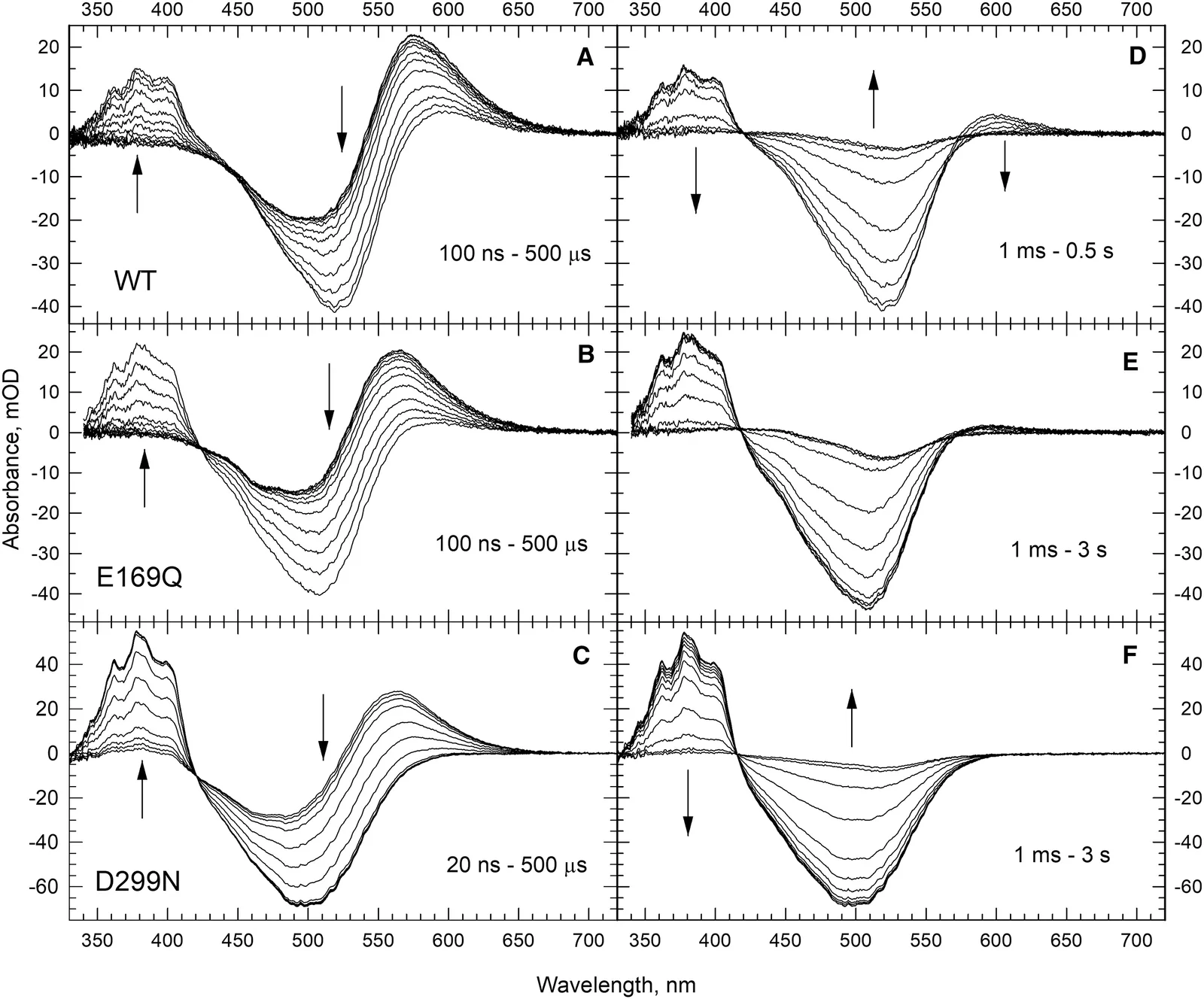
SVD-filtered TROD spectra of the WT, and the E169Q and D299N variants recorded in the 100 ns–0.5 s, 100 ns–3 s, and 20 ns–3 s delay time ranges, respectively, at values 1, 2, and 5 in each decade of time. Arrows indicate the direction of absorption change as the delay time progresses. The data are separated into two groups following the rising (*A*–*C*) and falling (*D*–*F*) trend of the deprotonated form at 380 nm at early and late times, respectively.

The TROD spectra for the WT, and for the E169Q and D299N variants of *Ca*ChR1 collected in the 100 ns–0.5 s, 100 ns–3 s, and 20 ns–3 s time-windows, respectively, at values 1, 2, and 5 in each decade of time, are displayed in Fig. 2. They are filtered by SVD and separated into two time-windows for easier viewing: Fig. 2, *A* and *D*, *B* and *E*, and *C* and *F*, for the WT, E169Q, and D299N, respectively. A few differences between the protein samples are obvious. The D299N variant shows more deprotonated chromophore, absorbing around 380 nm, than the WT and E169Q variant, and displays the smallest absorption around 600 nm, decaying at late times (Fig. 2, *D*–*F*). As seen at early times (Fig. 2, *A*–*C*), it also has the smallest blue-shifted form that narrows the trough of the WT difference spectra at 500 nm. Common for the proteins is the relatively small absorbance change in the early ms time range, which appears as a congregation of absorption traces (Fig. 2, *E* and *F*).

### Global exponential fitting

**Figure 3:**
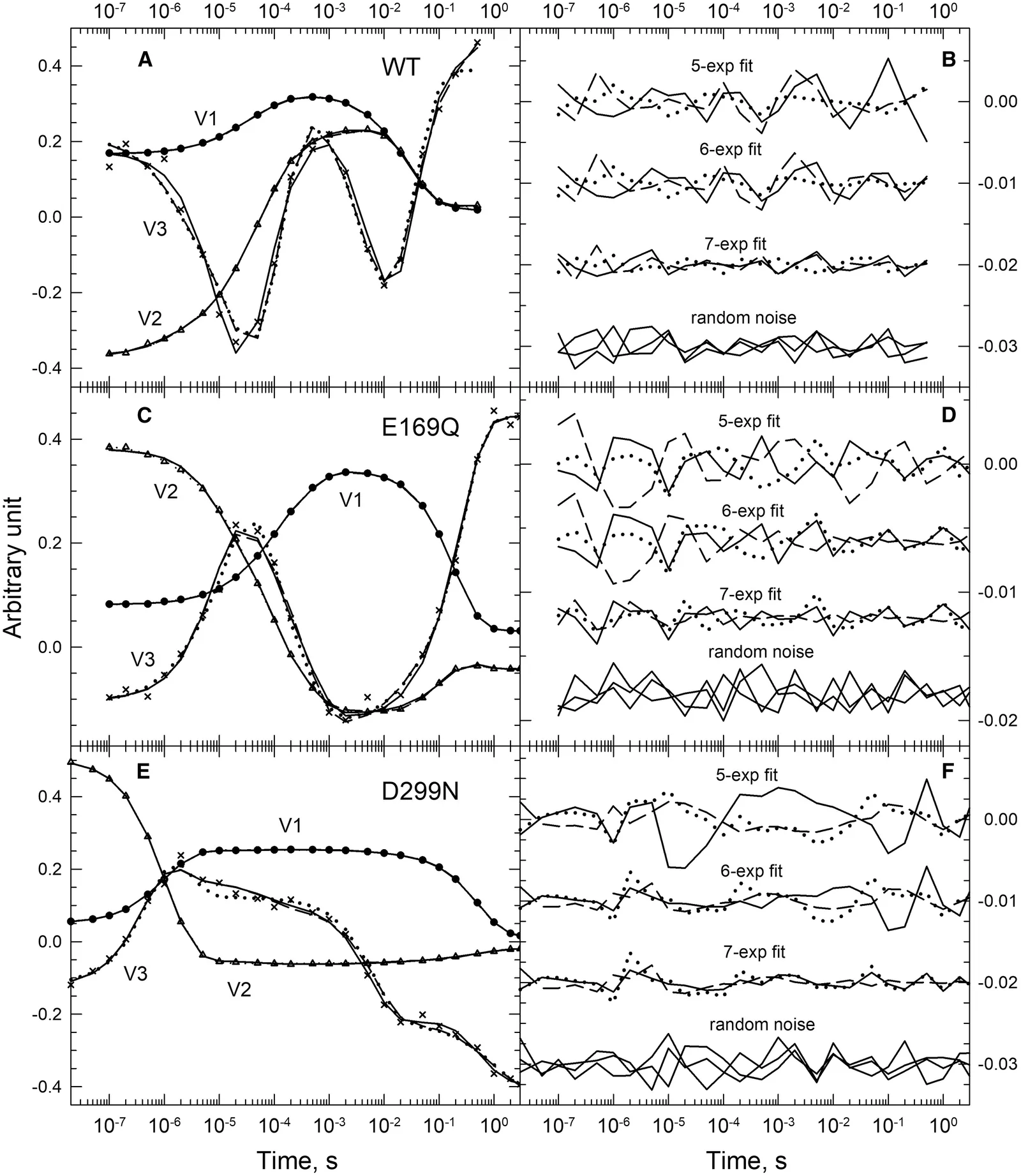
(*A*, *C*, and *E*) Exponential fit to the first 3 significant V-vectors using 5 (*dot*), 6 (*dash*), and 7 (*solid*) exponentials. (*B*, *D*, and *F*) comparing the residuals of the exponential fits to the random noise level. Based on the amplitudes and the lag 1 correlation coefficients calculated for the residuals, the results of the 7-exponential fit are chosen for further analysis.

To get more insight into the kinetics, the data sets were fitted to sums of exponential functions. Before the fitting process, the data sets were subjected to SVD analysis. Similar to the anion channelrhodopsin *Gt*ACR1 (32), SVD found only a limited number of independent spectral (U) and temporal (V) vector pairs contributing to the data above 1–2%. The significance values were 1.33, 0.54, 0.03, and 0.01 for the WT; 1.49, 0.54, 0.06, 0.02 for the E169Q variant; and 3.54, 0.68, 0.09, and 0.02 for the D299N variant. Thus, the data sets are practically reproduced by the most significant three to four U-V vector pairs, and thus one would expect the V-vectors to be adequately fitted with no more than four exponential functions. This is not the case. As shown in Fig. 3, *B*, *D*, and *F*, even the five-exponential fits to the three most significant V-vectors for all three proteins show residuals exceeding the noise level in most parts or in the entire time range. Moreover, the autocorrelation coefficients for many of the residuals are above 0.2. Adding one more exponential makes some improvement, but not in the entire time range and not for all three proteins. For consistency of the kinetic analysis, the seven-exponential fits are used for all of them. The seven lifetimes for the WT are: 0.68, 11, 65, and 390 *μ*s, 6.7, 32, and 220 ms; for E169Q: 1.2, 16, 96, and 470 *μ*s, 15, 110, and 230 ms; for D299N: 0.49, 1.5, and 63 *μ*s, 5.8, 74, and 540 ms, and 3.0 s. Note that the lifetimes derived in a fit that involves seven non-orthogonal exponential functions inevitably possess relatively high uncertainties, especially those that belong to b-spectra with small amplitudes. The number of exponentials and their accurate values are undoubtedly important factors in all kinetic analyses aimed at deriving the mechanism and the microscopic reaction rate constants. There, underfitting and overfitting the data, or using inaccurate lifetimes, may cause severe complications. That kind of analysis, however, is not part of this work. Our goal is to deduce the spectral forms and their time evolution in the kinetics and, for that, the uncertainty in lifetimes has little if any importance; thus, the seven-exponential fit serves our goal perfectly.

The discrepancy between the low number of independent U-V vector pairs and the high number of exponentials required to fit the few V-vectors is a clear sign of isospectral intermediates being present in the kinetics. Generally, isospectral intermediates are difficult to handle, and arranging them in a single reaction chain long enough to accommodate the large number of lifetimes is often impossible. It is more realistic to consider that such intermediates belong to shorter, parallel reaction paths functioning simultaneously (33). Untangling parallel reaction kinetics is a formidable task, and we make no attempt to do that in this work.

**Figure 4:**
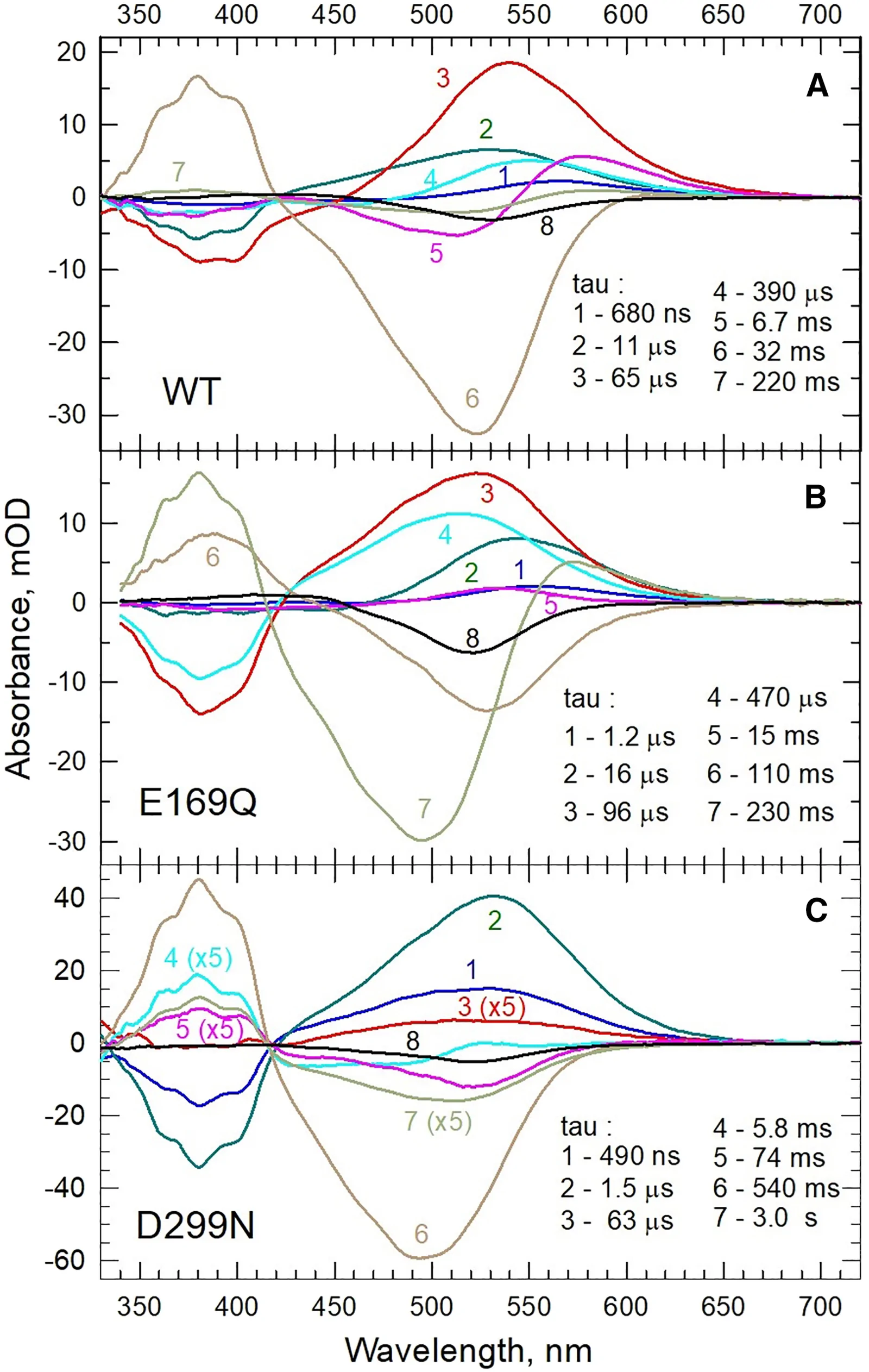
b-Spectra and decay times of the 7-exponential fit for the WT (*A*), and the E169Q (*B*) and D299N (*C*) variants, respectively. The traces are smoothed for clarity. The low amplitude bs3, bs4, bs5, and bs7 spectra of D299N are expanded by a factor of 5 for better viewing.

The b-spectra and the corresponding lifetimes found for the WT and the counterion variants are displayed in Fig. 4. Usually, the b-spectra are the primary source of information for the derivation of molecular mechanisms and, in most cases, can be interpreted with relative ease: the positive peaks belong to the decaying spectral forms and the negative troughs represent those emerging during the transition (34). For reasons explained below in more detail, we use the term *spectral form* instead of the more familiar *intermediate*. Eight b-spectra, representing seven transitions between five spectrally different intermediates, present a picture too complex to handle. Without additional supporting information from other sources, it is practically impossible to make connections between the b-spectra and the potential reaction steps in a scheme. A few trends in the kinetics, however, can be deduced from the b-spectra in Fig. 4. Because of better spectral and time resolution, spectral changes on the submicrosecond timescale, missed by single-wavelength recording, were detected. As seen in the WT, and less clearly in the E169Q variant, deprotonation of the Schiff base, combined with other changes, occurs gradually in the ns to early ms time range (*A* and *B*). It is followed by recovery of the protonated chromophore in two steps with very different relative amplitudes for the two proteins. The WT recovers practically in the faster step with 32 ms lifetime, while, in the E169Q variant, the recovery is shared almost equally between the closely spaced 110- and 230-ms steps. In the D299N variant, the Schiff base deprotonates very early, with 0.49- and 1.5-*μ*s lifetimes (*C*). It is followed by protonation steps having very small amplitudes; thus, the corresponding b-spectra, bs3, bs4, bs5, and bs7, are expanded by a factor of 5 for better viewing. Most of the protonated Schiff base recovers with the 0.54 s lifetime. Another feature that distinguishes the D299N variant from the WT and the E169Q variant is the lack of absorption in the 550–600 nm range of the b-spectra corresponding to the late recovery steps. None of the b-spectra shows spectral evidence of an M-to-O transition.

While the trends in the b-spectra are described with relative ease, interpreting b-spectra quantitatively, in terms of decaying and emerging intermediate fractions necessary to derive a mechanism, can be challenging. This is especially true for b-spectra produced by multistep chains in parallel reaction paths, which may well apply to our data. Because of this, the b-spectra will not be further analyzed in this work.

### Sequential intermediates, intermediate states, and spectral forms

The term *intermediate* commonly refers to a member in a kinetic scheme, irrespective of the validity of the proposed scheme, and will be used in that sense here too. Intermediates derived from experimental data using a proposed scheme, such as the popular unidirectional sequential scheme, are not necessarily single molecules, as demonstrated below. The term *intermediate state* we reserve for intermediates that are single molecules, i.e., *molecular states* seen in proven and thus valid mechanisms. These states have unique spectral characteristics that can be defined without knowing the mechanism itself. The term *spectral form* will refer to such a spectrum. In a kinetic analysis based on UV-vis spectra, such as used in this work, a spectral form may represent more than one intermediate state because intermediates with different conformations but practically identical UV-vis spectra cannot be distinguished.

**Figure 5:**
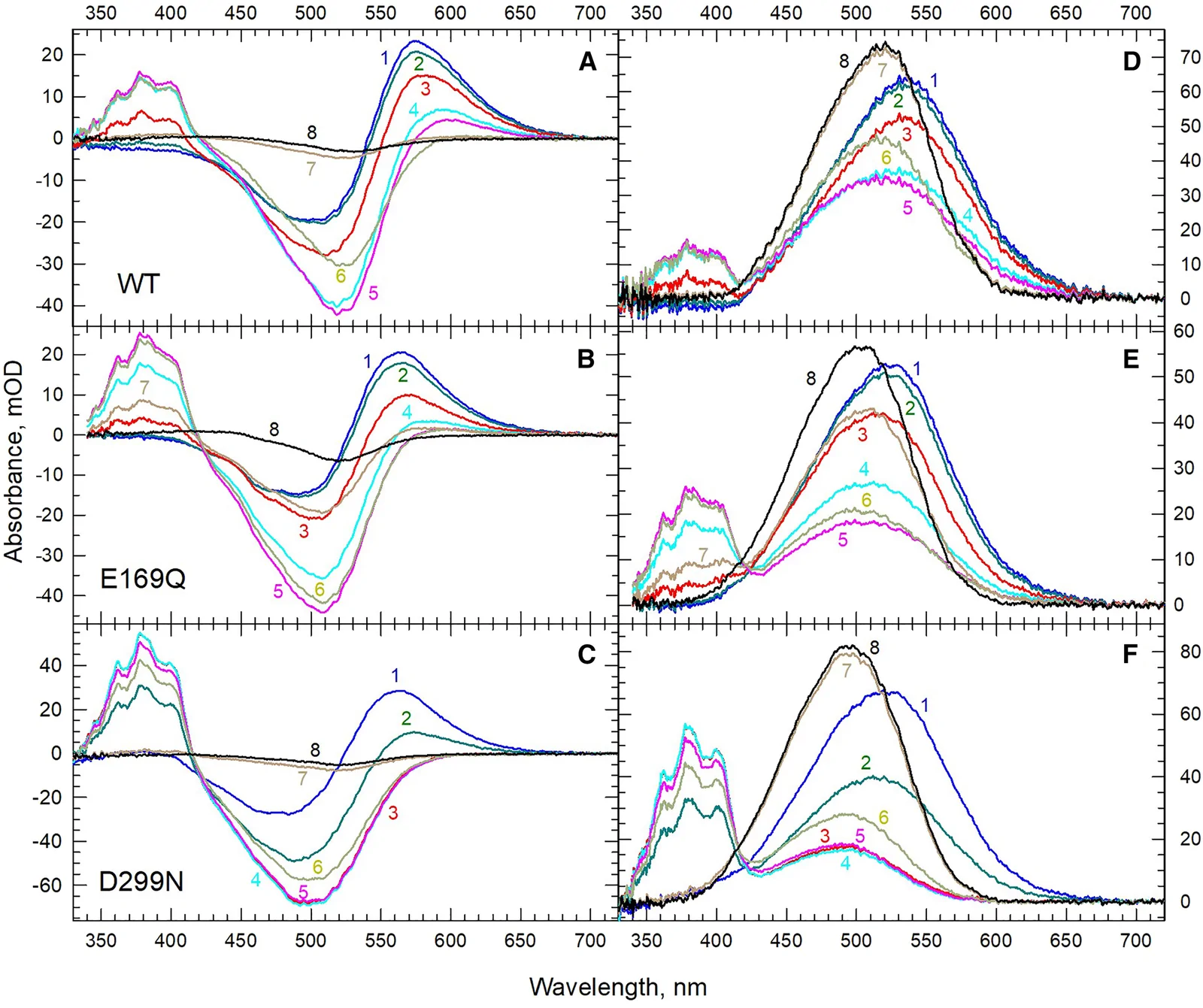
(*A*–*C*) Sequential intermediate difference spectra, In1-In8, calculated from the b-spectra assuming a unidirectional scheme in which the intermediates decay by the apparent rates (inverse of lifetimes). (*D*–*F*) Absolute spectra of sequential intermediates obtained by adding back appropriate amounts of bleach as described in the text. Note that Ina2-Ina7 are all composite spectra and unsuitable for spectra of true reaction intermediates.

Our goal is to derive the spectral forms involved in the *Ca*ChR1 kinetics and, for that, the spectra of the sequential intermediates are a better choice than the b-spectra discussed above (32). These are calculated from the b-spectra and are displayed in Fig. 5, *A*–*C*. We have to emphasize that the sequential scheme used in this work is *not* the reaction mechanism of *Ca*ChR1, and the apparent rates, corresponding to the seven lifetimes obtained in the exponential fit, are *not* real reaction rate constants. The scheme is used only as a convenient tool in the spectral analysis below.

Constructing the eight-member sequential scheme merely cuts the unknown reaction mechanism into eight stages, called sequential intermediates, separated by the seven lifetimes. At each stage, all the true intermediate molecular states that are present contribute to the spectrum corresponding to that particular lifetime, regardless of what real reaction chains in the unknown kinetic mechanism they belong to. The spectral contributions are congregated into one pool called the sequential intermediate spectrum. Thus, most of the sequential intermediate spectra are not expected to be single spectral forms, molecular states capable of controlling, for instance, channel opening and closing. Instead, they are weighted sums of spectral forms referenced against the dark spectrum bleached by the laser. This guiding principle is followed in the spectral deconvolution described below.

### Spectral deconvolution of sequential intermediates

**Figure 6:**
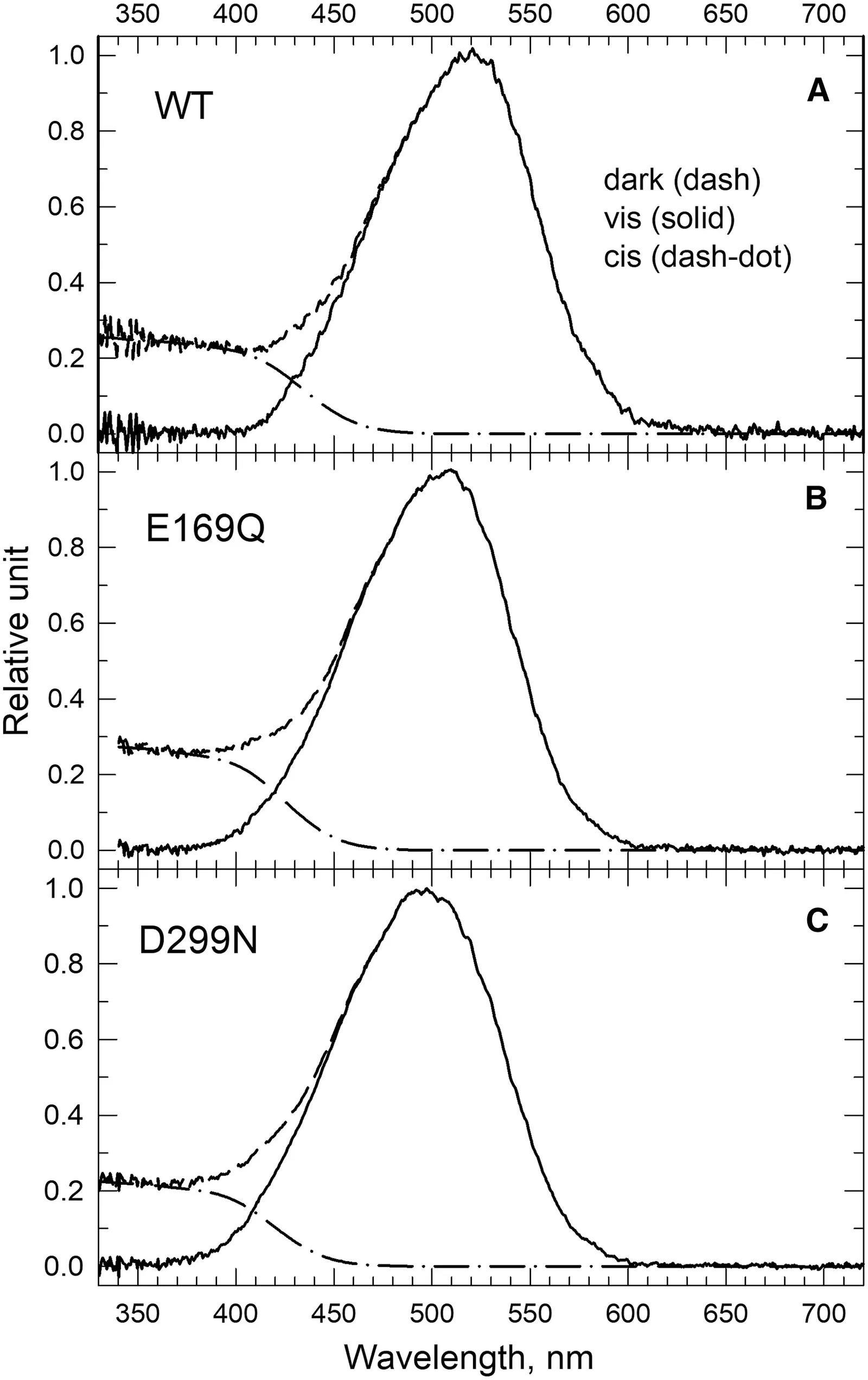
Separation of the visible (*solid*) and *cis* (*dash-dot*) bands in the dark spectra (*dash*) of the WT (*A*), the E169Q (*B*), and D299N (*C*) variants.

Because the sequential intermediate difference spectra in Fig. 5, *A*–*C*, represent different stages of the spectral evolution, they are very similar in shape to the TROD spectra shown in Fig. 2 and carry basically the same kinetic information. The deconvolution of intermediate spectra into spectral forms is done more conveniently by using not the difference but the more familiar absolute spectra shown in Fig. 5, *D*–*F*. They were produced by adding back the bleached dark spectrum to the difference spectra. For accurate spectral manipulations in the analysis, the dark spectrum and the TROD spectra were recorded on the same instrument. The amount of bleach is handled here in the following way. In addition to demanding positive absorbance values for each absolute spectrum, we also demand that the absolute spectra of all sequential intermediates must accommodate one, or a weighted sum of potential spectral forms. As mentioned above, a spectral form is considered to represent a molecular state and thus must have a set of spectral characteristics. The acceptable spectral forms for retinal proteins are defined by the concept of similar spectral shapes introduced by us earlier (35). Accordingly, any spectrum derived by shifting the visible band of the dark spectrum on the energy scale is an acceptable spectral form for a molecular state. This concept proved to be effective in the kinetic analysis of retinal proteins, including channelrhodopsins (32,35), and bovine and human rhodopsins (36,37). Before applying the concept, the dark spectrum has to be separated into the so-called *cis* band and the visible band. The spectral separation is shown in Fig. 6 for the three proteins. The *cis* band remains practically unchanged during the photoreaction and thus vanishes from the TROD records. The absolute spectra of sequential intermediates in Fig. 5, *D*–*F*, were obtained by adding back appropriate amounts of the visible band bleached by the laser light, so they do not include the *cis* band either.

Out of the eight intermediate spectra, Ina1 and Ina8 are considered to be spectral forms. Ina1 is analogous to the spectrum of the K intermediate of *Hs*BR, which is regarded as a molecular state, and Ina8 is the recovered form, R, spectrally very close to the bleach. Ina1 is slightly broader than the bleach shifted on the energy scale and has the typical red tail seen for all K intermediates of retinal proteins. Careful examination of Ina1 obtained for the D299N variant reveals that it already has a small contribution in the 350- to 420-nm region by the deprotonated chromophore, the M analog of *Hs*BR formed earlier than 20 ns, and which should be removed to get the proper K spectrum. The spectrum of the deprotonated M form of retinal proteins is usually well separated from the spectra of the other intermediates; its shape can be reproduced by sums of Gaussian components and used as the M spectral form. The Ina2-Ina7 intermediate absolute spectra in Fig. 5, *D*–*F*, are composite spectra having both deprotonated and protonated forms present simultaneously. These spectra belong to multiple molecular states, confirming that the straight sequential scheme does not represent the true kinetic mechanism.

Having the K and R spectral forms taken from the experimental data directly, and combining them with the M spectral form, put severe restrictions on the shapes and amplitudes of the additional spectral forms potentially present in the composite spectra. Following the familiar *Hs*BR nomenclature, we name a spectral form closely spaced to the dark spectrum L, a blue-shifted one N, and a red-shifted one O. While the amplitudes of the K and R spectral forms are predetermined, that of M is more flexible. This offers some leverage over the amplitudes of the other extracted spectra. If present, the L spectral form is best extracted from the early intermediates, Ina2-Ina3, while the N and O spectral forms from the later ones, Ina4-Ina6. The L and R spectral forms are expected to be closely spaced, and thus hard to separate properly in the deconvolution. Logically, the recovered R form is absent at early times but dominates the very late intermediate spectra.

In the deconvolution of sequential intermediate spectra, the contributions made by the known K, M, and R spectral forms are estimated and subtracted from the spectra first. Then, the remaining spectra are normalized by their fraction values using the formula: *SP=(Ina–a*×*K–b*×*M–c*×*R)/(1–a–b–c)*, where *a*, *b*, and *c* are the contributions made by K, M, and R, respectively. Each remaining spectrum is compared with the bleach shifted on the energy scale, or to combinations of shifted spectra if the remaining spectrum appears to be composite. This process is repeated until meaningful and consistent results are obtained. Composite remaining spectra complicate the deconvolution somewhat because several sequential intermediate spectra, which may contain the potential spectral forms, have to be evaluated simultaneously to get consistent results. While the deconvolution can be carried out using either difference or absolute spectra, the latter offers a few practical advantages.

**Figure 7:**
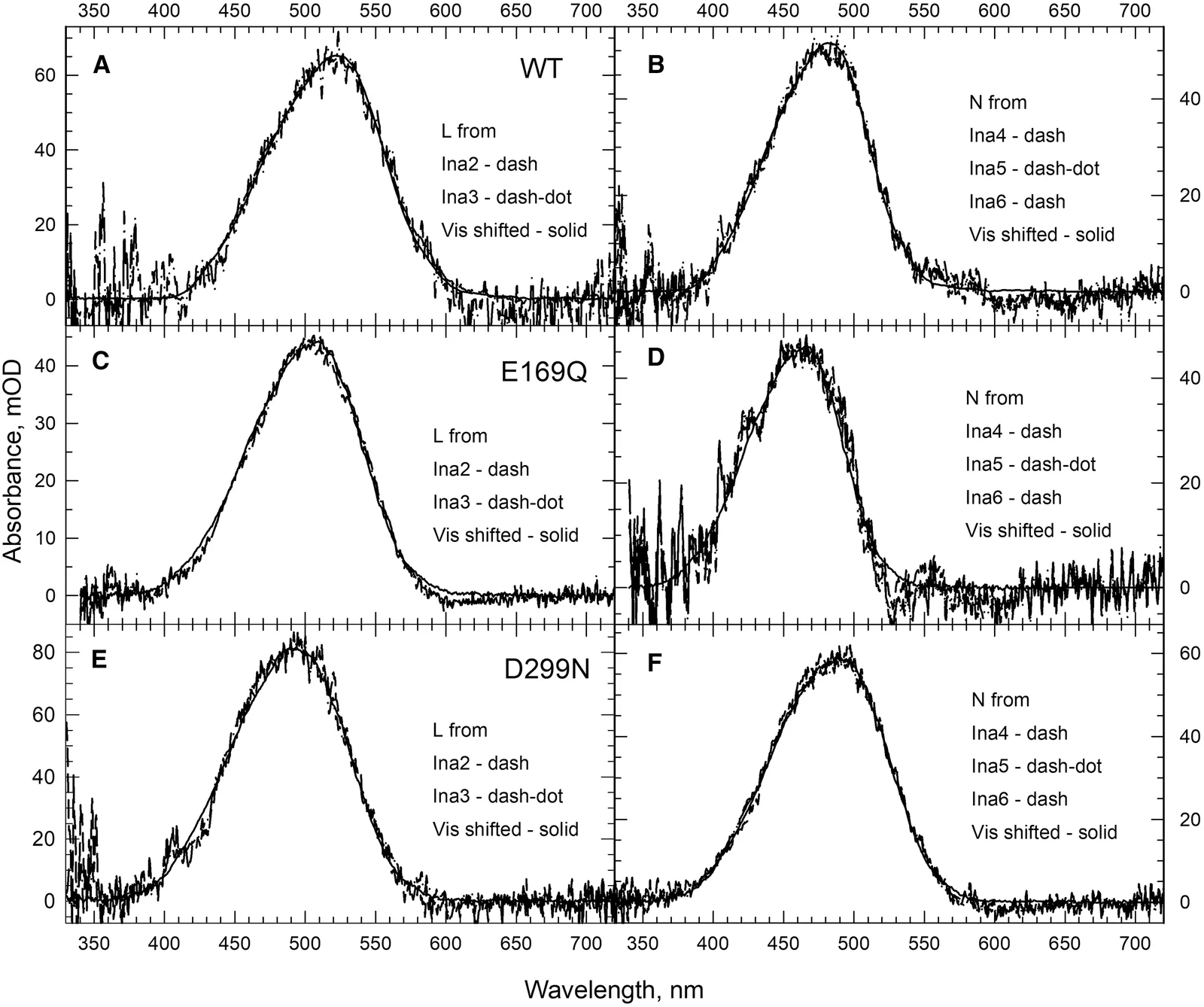
Deconvolution of the L spectral form from Ina2 and Ina3 of the WT (*A*), and the E169Q (*C*) and D299N (*E*) variants. Deconvolution of the N spectral form from In4, Ina5, and Ina6 of the WT (*B*), and the E169Q (*D*) and D299N (*F*) variants. Solid lines are the bleach spectra shifted on the energy scale to match the corresponding deconvoluted spectra.

The results of the deconvolution are shown in Fig. 7 for the WT (Fig. 7, *A* and *B*), the E169Q (Fig. 7, *C* and *D*), and the D299N (Fig. 7, *E* and *F*) variants. The L spectral forms deconvoluted from the Ina2 and Ina3 absolute spectra are shown in Fig. 7, *A*, *C*, and *E*. Also displayed are the L forms produced by shifting the corresponding bleach on the energy scale (*solid lines*). Fig. 7, *B*, *D*, and *F*, show the N spectral form deconvoluted from Ina4, Ina5, and Ina6, and also the N produced by blue-shifting the bleach on the energy scale (*solid lines*). There was no obvious sign for additional spectral forms being present, such as a red-shifted O-like noticeably different from the K-like, as suggested (20). Apart from the noise, the extracted L and N spectral forms match the shifted bleach spectra surprisingly well, signifying the success of deconvolution. The fraction values of all spectral forms present in the sequential intermediates of the WT, the E169Q, and D299N variants are in Tables 1, 2, and 3, respectively. Note that the L and N spectral forms appear together in most of the sequential intermediate spectra, indicating that they were deconvoluted simultaneously. The random noise in the L and N spectral forms in Fig. 7 is due mainly to the low fraction values. The noise levels of the L and N spectral forms, extracted from those sequential intermediates that are not displayed in the figure, were often too high for presentation. The small discrepancies are due, most likely, to recording errors. They are more enhanced at the blue end of the spectral range due to the rapidly decreasing sensitivity of the recording system toward the UV range.

**Table 1:** WT: K, L, M, N, and R spectral form content of sequential intermediates In1-In8. K assigned to O is in parentheses

	K	L	M	N	R
In1	1.00	–	–	–	–
In2	0.915	0.05	0.023	0.012	–
In3	0.78	0.04	0.15	0.03	–
In4	0.52 (0.10)	0.03	0.295	0.155	–
In5	0.40 (0.40)	0.03	0.32	0.19	0.06
In6	0.13 (0.13)	0.005	0.275	0.105	0.485
In7	0.04 (0.04)	–	0.015	–	0.945
In8	–	–	–	–	1.00

**Table 2:** E169Q: K, L, M, N, and R spectral form content of sequential intermediates In1-In8

	K	L	M	N	R
In1	1.00	–	–	–	–
In2	0.90	0.085	0.01	0.005	–
In3	0.60	0.25	0.09	0.06	–
In4	0.355	0.175	0.39	0.08	–
In5	0.25	0.11	0.56	0.08	–
In6	0.22	0.10	0.52	0.09	0.07
In7	0.20	0.10	0.19	–	0.51
In8	–	–	–	–	1.00

**Table 3:** D299N: K, L, M, N, and R spectral form content of sequential intermediates In1-In8

	K	L	M	N	R
In1	0.93	–	0.07	–	–
In2	0.465	0.085	0.45	–	–
In3	0.02	0.12	0.75	0.11	–
In4	0.01	0.07	0.75	0.17	–
In5	0.02	–	0.69	0.29	–
In6	0.03	–	0.58	0.24	0.15
In7	–	–	0.025	0.025	0.95
In8	–	–	–	–	1.00

**Figure 8:**
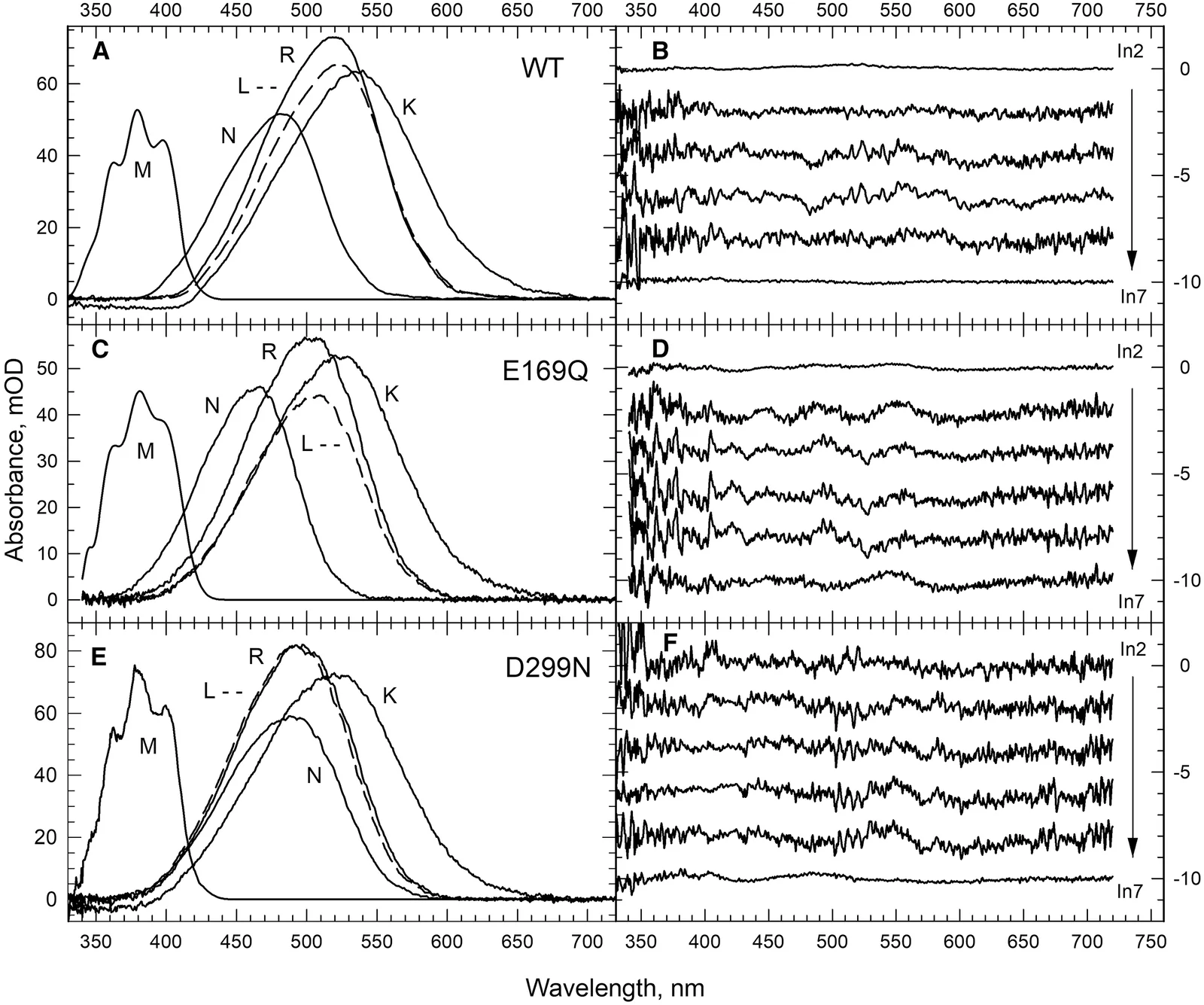
Spectral forms present in the kinetics of the WT (*A*), the E169Q (*C*), and D299N (*E*) variants. The K and R forms are from experiment, the M is a sum of Gaussian components to mimic the shape of the blue end of experimental spectra, and L and N are the bleach spectra shifted on the energy scale. (*B*, *D*, and *F*) The residuals of reproduction of the sequential intermediate spectra, In2-In7, for the WT, and the E169Q and D299N variants, respectively. The traces are shifted by 2 mOD for clarity. The residuals are calculated as the difference between the experimental spectra and the spectra reproduced by the weighted sums of the K, L, M, N, and R spectral forms according to the corresponding composition matrices derived in the deconvolution.

The K, L, M, N, and R spectral forms present in the sequential intermediate spectra are displayed in Fig. 8, *A*, *C*, and *E*. As mentioned, the K and R forms are taken directly from the experiment, and the M form is a compromise between the experimental and simulated shapes. The magnitude of M was adjusted until the amplitudes of the L and N spectral forms reached meaningful levels in the deconvolution. The L and N spectral forms shown in the figure are the ones produced by shifting the bleach on the energy scale and drawn by solid lines in Fig. 7. Using the matrix of the *m* spectral forms, *E(λ,m)*, and the corresponding composition matrices from Tables 1, 2, and 3, *Q(m,n)*, the sequential intermediate spectra, *In(λ,n)*, for the WT, the E169Q and D299N variants were reproduced: *In(λ,n) = E(λ,m)*_*^∗^*_ *Q(m,n)*. Due to the nearly perfect deconvolution, the experimental and reproduced spectra are too close to distinguish, thus the differences between them are plotted in Fig. 8, *B*, *D*, and *F*, shifted by 2 mOD for clarity of viewing. The discrepancies are small, ±1 mOD, and are within the range of recording errors.

### Spectral forms and their time evolution

The absorption maxima of the derived spectral forms for the WT, the E169Q, and the D299N variant proteins are shown in Table 4. The recovered R spectral forms for the three proteins are only 1–2 nm blue-shifted from the bleach spectra shown in Fig. 6. As mentioned, the red-shifted K spectral forms are slightly broader than the bleach spectra shifted on the energy scale. The shifts were estimated to be 17 nm for the WT and E169Q variant, and 22 nm for the D299N variant. The main peak of the deprotonated M form is located at 380 nm for all three proteins. The L spectral forms are spaced close to the bleach spectra, 2 nm red-shifted for the WT, none for E169Q, and 4 nm blue-shifted for the D299N variant. The N spectral forms are all blue-shifted relative to the bleach, by 38 nm for the WT, by 42 nm for the E169Q, and by only 10 nm for the D299N proteins.

**Table 4:** Absorption maxima of the spectral forms

	WT	E169Q	D299N
Dark	521	507	497
K	538	524	519
L	524	507	493
M	380	380	380
N	483	465	487
R	520	505	496

The question of how unique the deconvolution results are is addressed briefly. As mentioned above, the amplitudes of L and N spectral forms and their fractions in the sequential intermediates depend on the magnitude of M chosen. Our aim was to bring the amplitudes of L, M, and N close together, and also close to the size of the K form, while maintaining consistency in the deconvolution for all sequential spectra. Applying this restriction, maintaining the balance of fractions and also the shapes and amplitudes of the spectral forms simultaneously in all six intermediate spectra deconvoluted, narrowed the range of possible solutions greatly and left little room for uncertainties. In addition, exploring the range of small uncertainties has little merit because these have little if any significance regarding the conclusions drawn from our analysis.

**Figure 9:**
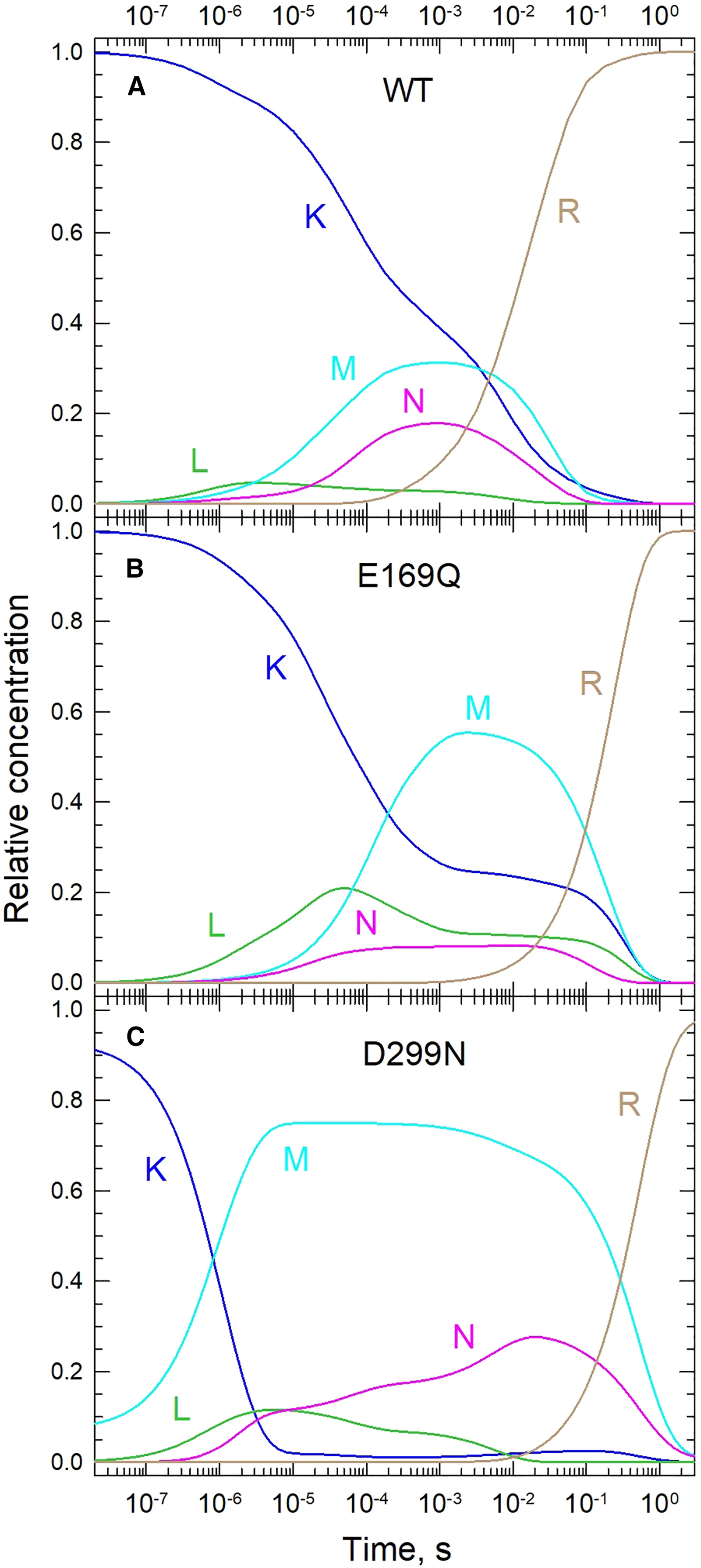
Temporal evolutions of the K, L, M, N, and R spectral forms present in the WT (*A*), and the E169Q (*B*) and D299N (*C*) variants.

Having extracted the spectral forms that belong to potential intermediate states in the kinetics, the time evolution of these *m* spectral forms can be deduced: *C(m,t) = Q(m,n)*_*^∗^*_ *C*_*in*_*(n,t).* Here, *Q(m,n)* is the composition matrix mentioned above and *C*_*in*_*(n,t)* is the time evolution of the *n* sequential intermediates calculated based on the kinetic matrix of the sequential scheme. The timescale used in the calculation is different from the experimental delay times, it is smoother and covers a broader range for better viewing. The time evolutions of the K, L, M, N, and R spectral forms are shown in Fig. 9, *A*–*C*, for the WT, the E169Q, and the D299N variants, respectively. It is evident that the WT completes the cycle faster than the mutated proteins, and it has less M accumulated, in agreement with the published results (20). It has also very little L but has the highest K level that decays in the late step(s). In the D299N variant, the K drops sharply, and there is very little left starting as early as 10 *μ*s. It is replaced by M and this variant shows the highest M level lasting until the very end. Because of their little overlap in time, the separation of L and R spectral form contributions poses no problems for the WT and the D299N variant, but it is somewhat more uncertain for the E169Q variant, where the overlap in time is bigger.

The many and often small changes in concentrations seen in Fig. 9 are not easily explained within the framework of a single reaction chain and call for introducing parallel paths composed of isospectral intermediates. Thus, some of the spectral forms may belong to more than one isospectral intermediate state and, consequently, some of the time evolution traces may represent the sum of two or more consecutively emerging intermediate states. These states can often be separated with the help of electrophysiological records, as reported for the anion channelrhodopsin *Gt*ACR1 earlier (33).

The kinetic and spectral differences between the WT and the variants are reflections of the differences in their structure. The absorption maximum of the dark spectrum is controlled by the retinal environment (opsin shift). The two carboxylic groups of amino acids E169 and D299 form a complex counterion to the protonated Schiff base (27). The D299 is ionized in the dark, and following photoexcitation, it quickly receives a proton from E169, which then becomes ionized (28). This counterion switching mechanism must be absent in the variants. Most likely the E169 and the D299 residues in the D299N and E169Q variants, respectively, are ionized in the dark and stay ionized following photoexcitation. Interestingly, the replacement of either counterion by a neutral amino acid shifted the dark spectrum to the blue. In our rough estimate, the shifts are 14 nm for the E169Q and as much as 24 nm for the D299N exchange. Better electrical shielding of the protonated chromophore and an increased ratio of the 13-*cis* to all-*trans* retinal can both induce a blue shift in the absorption spectrum. Regardless of the origin of the shift, the replacements changed the retinal environment in both variants and more significantly in the D299N protein. It is not totally unexpected that the changes in the retinal environment are coupled to changes in the protein conformation, which then lead to photocycle kinetics different from the WT, as the ones shown in Fig. 9. The channel currents published for the WT, and the E169Q and D299N variants (20), affirm the conformational kinetic differences: compared with the WT, the E169Q protein shows current amplitudes reduced by a factor of 15–20, and the D299N an even bigger, by a factor of 25–30, current reduction. Photoexcitation is often followed by fast internal proton and dipole movements in the protein which can induce large current signals in the measuring circuits at early times. Because of this, the whole current signal is available only for the WT protein (20).

For the WT protein the time-dependent absorption of the M form, measured at 390 nm with the single-wavelength detection technique, reportedly followed the time course of the current decay reasonably well (20), implying that the M form may be associated with the channel function. In single-wavelength experiments, the absorption of the M form is easily and separately traced from the others. Thus, only the M form was compared with the current trace.

### Searching for the conductive state and the pitfall of the single-wavelength approach

The most common experimental technique in kinetic studies is the recording of absorption changes at selected wavelengths as a function of time. The wavelengths are chosen according to the absorption range of the presumed intermediates. In case of retinal proteins these are the analogs of the known *Hs*BR intermediates. It is often believed that the single-wavelength traces, which can be fitted to sums of exponential functions with relative ease, give an accurate and very straightforward picture of the kinetic changes. Having derived the spectral forms, the spectra of potential intermediates and their evolution in time, we are in the position to evaluate the single-wavelength approach in a quantitative way.

**Figure 10:**
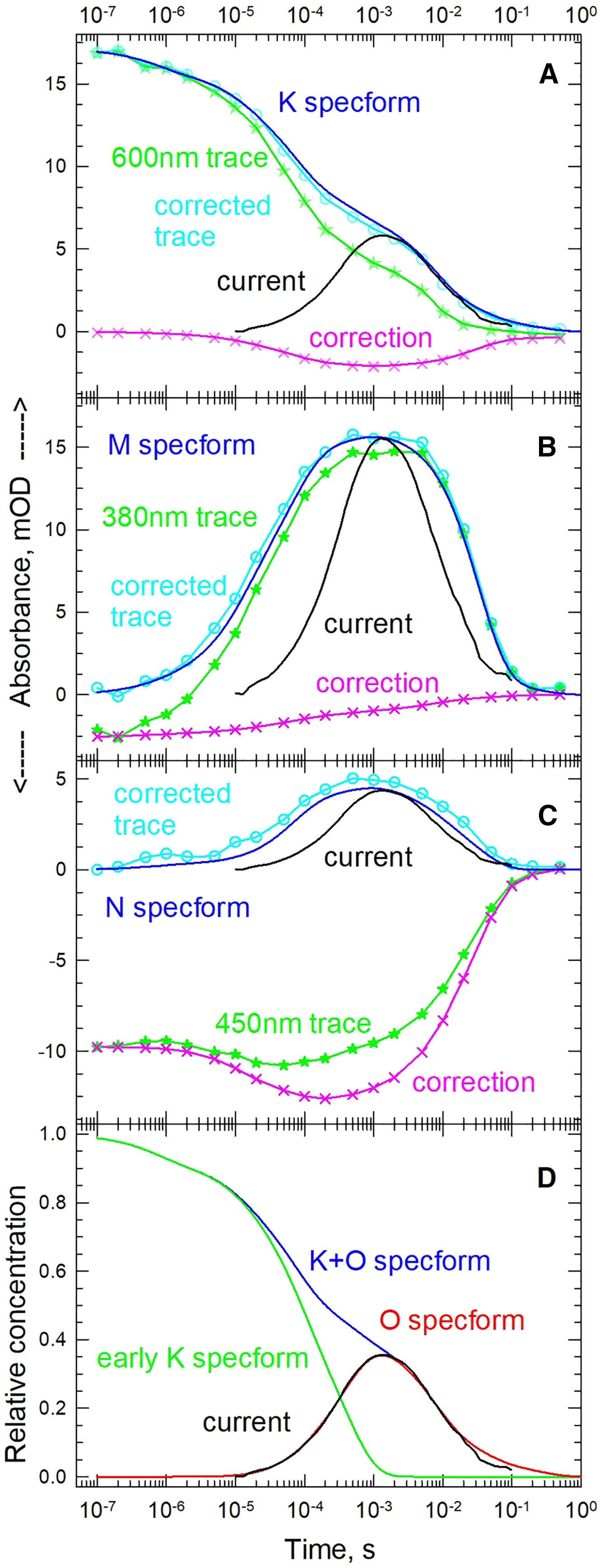
(*A*–*C*) Comparison between the single-wavelength traces taken from the experimental data at 600, 380, and 450 nm and the time evolution of the K, M, and N spectral forms derived for the WT protein, respectively. The discrepancies can be removed by applying the necessary corrections to the experimental traces. (*D*) Separation of the K spectral form (K + O, *blue*) of the WT protein into early K (*green*) and late O (*red*) components. The good agreement between the O spectral form and the channel current (*black*) traces shows that the O spectral form represents the intermediate(s) responsible for the channel opening.

Above, we established that the K, L, M, N, and R spectral forms are involved in the kinetics and, based on their time evolution, we consider the K, M, and N to be the best candidates for the open channel state in the WT protein. The difference spectra of the candidates suggest that traces taken at 600, 380, and 450 nm would be a good selection to mimic the single-wavelength traces recorded for the WT protein. These traces can be compared with the time evolution curves of the corresponding spectral forms deduced in the analysis above, and also with the time-resolved current published (19). The latter was digitized at selected delay times for the comparison shown in Fig. 10, *A*–*C*. The blue lines are the spectral form evolutions, the green lines with stars are the traces taken from the experimental data matrix by averaging in a 4-nm spectral range, and the black lines represent the current. While there is very little overlap between the absorption spectra of the K and the other spectral forms at 600 nm in Fig. 8, the single-wavelength and the spectral form traces in Fig. 10 *A* deviate significantly. The same applies to the 380-nm trace in Fig. 10 *B*, and the 540-nm trace in Fig. 10 *C*, which does not even resemble that of the N spectral form evolution. In addition, the 600-nm trace in Fig. 10 *A* shows practically no decay in the time range that belongs to the two longest lifetimes found in the global fit above.

These disagreements are not caused by any error in the analysis presented; it is due to a pitfall present in the interpretation of the single-wavelength traces. A trace at the wavelength where the intermediate absorbs, and no other intermediate contributes to it, is often considered to reflect the kinetics of the selected intermediate accurately. This is an incorrect assumption in every situation where the bleach spectrum overlaps with that of the selected intermediate. The reason for this is that the recorded absorption trace at every wavelength contains the entire amount of the bleach missing, not just the amount that belongs to the intermediate in question. While the missing bleach in the trace of N at 450 nm in Fig. 10 *C* is obvious, the presence of the extra bleach in the 600- and 380-nm traces is not. The extra bleach, which follows the kinetics of the other intermediates, modifies the true absorption trace of the selected intermediate, introduces extra decaying and rising exponentials as seen in the 380-nm trace, or cancels the real ones as in the 600-nm trace. Thus, changes that have nothing to do with the evolution of the selected intermediate could be mistakenly assigned to it. The effect of the extra bleach can be substantial when the fraction of the selected intermediate in the total population is low. All these problems are very clearly seen in the figure.

Of course, the time-dependent extra bleach can be calculated, as shown by the pink lines with crosses in the figure, and be removed to yield the corrected traces, as shown by the cyan lines with circles in the figure. However, to do that it is necessary to know the difference absorption spectra and the time evolution of all the intermediates involved in the reaction before they are identified. This is the catch 22 of the single-wavelength approach.

Because of the pitfall described above, the current trace should be compared with the time evolution of the spectral forms and not with the single-wavelength traces. The decay of the current is well matched by the decay of the K spectral form, as shown in Fig. 10 *A*. The rise and fall of the current and the M spectral form show big differences, thus the latter seems to be the least likely candidate for the open channel state. The differences for the N spectral form are somewhat smaller but still quite significant. As reported (19), the current levels displayed by the E169Q and D299N variants are much lower than that of the WT. The K spectral form is practically missing in the D299N variant after 10 *μ*s, which strongly supports the channel assignment to the K spectral form. While the late kinetics of the K spectral form for the E169Q variant is in agreement with the long-lasting current trace, its level is somewhat higher than expected for the relatively small current level. The disagreement, however, can easily be explained by smaller channel conductance in this mutant. The traces for the M and N spectral forms in Fig. 9, *B* and *C*, are hard to reconcile with the current kinetics.

### Proposing the O intermediate state for the open channel state

Based on these arguments, the K spectral form present in the late sequential intermediates is proposed to represent the intermediate state that controls the channel opening and closing, and to be the channel state. It is not very rational to assume that the K intermediate present at the earliest times in the reaction remains unchanged and becomes the open channel state at late times. It is far more realistic to propose that the K intermediate converts into another red-shifted intermediate in a spectrally silent transition and the channel opening is controlled by that new intermediate. Thus the long-lasting trace of the K spectral form of the WT protein, blue curve named K + O in Fig.10 *D*, must be separated into two: an early part that is not associated with the channel, and a late part that is assigned to the conductive intermediate state, or states, named O. The entire amounts of the K spectral form in the In5-In7 sequential intermediates in Table 1 are assigned to the O conductive state. The rising part of the current is accounted for by assigning an additional amount of the K spectral form present in In4 to the O state, 0.1 out of 0.52 K form. These assignments, enclosed in parentheses in Table 1, yielded a nearly perfect reproduction of the entire current trace (*black line* in Fig. 10 *D*) by the time evolution of the O intermediate state (*red curve* in Fig. 10 *D*). The remaining early K spectral form decays completely at 1 ms (*green curve* in Fig. 10 *D*). Note that the conductive O state in Table 1 stretches over four late sequential intermediates, from In4 to In7, which clearly shows that associating a sequential intermediate with the open channel state is not necessarily a good choice and may lead to problems in the interpretations of kinetic results. Interestingly, cutting the K spectral form into nonconductive and conductive parts makes *Ca*ChR1 similar to the anion channelrhodopsin *Gt*ACR1 (33).

A single path that includes all the spectral forms will not account for the seven exponentials in a meaningful way, so multiple parallel kinetic paths, including conductive and nonconductive ones, should be invoked. Each of these will contain isospectral intermediate states similar to the complex schemes reported for the anion channelrhodopsin. It is unlikely that the kinetics will have a unique solution. Solving it in a quantitative way is not part of our primary objective and will not be pursued here.

The backbone of the potential conductive cycles should include the K, O, and R intermediates, and the dark state D. Because of the two-step decay of the O spectral form, the mechanisms are likely to include two O intermediates. These may be arranged sequentially or in parallel in a single chain, or in two parallel chains. To propose conductive cycles that will reproduce the current signals measured in both laser and long-pulse experiments requires additional studies.

### Conclusion

The photocycle kinetics of *Ca*ChR1 was investigated by analyzing TROD spectra recorded in the nanosecond-to-second time window for the WT, and the E169Q and D299N variants. The results of SVD and global exponential fitting indicate the presence of isospectral intermediates in the kinetics (Table 4). For all three proteins, the sequential intermediate spectra deduced from the global exponential fitting were deconvoluted into the K, L, M, N, and R spectral forms representing potential intermediate states in the kinetics. The time evolutions of these forms were derived and compared with the published current traces. Based on the current trace published for the WT and the current levels reported for the variants, the K spectral form is proposed to represent the open channel state. The long-lasting K spectral form is assigned to a conductive state, named O, at late times. We propose that channel opening and closing in *Ca*ChR1, in the cation *Ps*ChR2, the viral cation channelrhodopsin OLPVR1 (38), and the anion *Gt*ACR1 channelrhodopsins are uniformly controlled by red-shifted intermediate states. The central role of the O state for gating in channelrhodopsins is in agreement with the mechanisms of the prototypical proton pump *Hs*BR at acidic pH (39), the sodium pumps KR2 (40,41) and *Er*NaR (42), and the chloride pump *Nm*HR (43,44), where the rise and decay of O states are linked to ion translocation reinforcing the relevance of the thermal *cis*-*trans* re-isomerization in the final stage of the photoreaction in these microbial rhodopsins (45).

## Data and code availability

Data will be shared upon reasonable request (iszundi@ucsc.edu).

## Acknowledgments

We thank Dorothea Heinrich and Kirsten Hoffmann (FU Berlin) for their help with the expression and purification of the membrane proteins, and Karoline-Luisa Lê Công (FU Berlin) for help with protein structure modeling. This work was funded by the 10.13039/501100001659German Research Foundation through EXC 2008/1 (UniSysCat) to J.H. and R.S., 390540038 by SFB 1078, projects B3 to J.H. and B4 to R.S. The support from 10.13039/100000002NIH (USA) grant R01 EY029343 to D.S.K. is also acknowledged.

## Author contributions

I.S. designed the research, analyzed the data, and wrote the first draft of the manuscript. V.M. and R.S. (and co-workers) prepared the samples. C.F. conducted the experiments. D.S.K., R.S., and J.H. initiated and coordinated the project. I.S., D.S.K., R.S., and J.H. revised the manuscript.

## Declaration of interests

The authors declare no competing interests.

## Declaration of generative AI and AI-assisted technologies in the writing process

During the preparation of this work the authors used DeepL and ChatGPT to edit the English style. After using this tool/service, the authors reviewed and edited the content as needed and take full responsibility for the content of the published article.
